# *Lactobacillus rhamnosus* L34 and *Lactobacillus casei* L39 suppress *Clostridium difficile*-induced IL-8 production by colonic epithelial cells

**DOI:** 10.1186/1471-2180-14-177

**Published:** 2014-07-02

**Authors:** Prapaporn Boonma, Jennifer K Spinler, Susan F Venable, James Versalovic, Somying Tumwasorn

**Affiliations:** 1 Interdisciplinary Program of Medical Microbiology, Graduate School, Chulalongkorn University, Bangkok, Thailand; 2 Texas Children’s Microbiome Center, Department of Pathology, Texas Children’s Hospital, Houston, Texas, USA; 3 Department of Pathology & Immunology, Baylor College of Medicine, Houston, Texas, USA; 4 Department of Microbiology, Faculty of Medicine, Chulalongkorn University, Bangkok, Thailand

**Keywords:** *Lactobacillus*, *Clostridium difficile*, Probiotic, IL-8, Anti-inflammatory

## Abstract

**Background:**

*Clostridium difficile* is the main cause of hospital-acquired diarrhea and colitis known as *C. difficile*-associated disease (CDAD).With increased severity and failure of treatment in CDAD, new approaches for prevention and treatment, such as the use of probiotics, are needed. Since the pathogenesis of CDAD involves an inflammatory response with a massive influx of neutrophils recruited by interleukin (IL)-8, this study aimed to investigate the probiotic effects of *Lactobacillus* spp. on the suppression of IL-8 production in response to *C. difficile* infection.

**Results:**

We screened *Lactobacillus* conditioned media from 34 infant fecal isolates for the ability to suppress *C. difficile*-induced IL-8 production from HT-29 cells. Factors produced by two vancomycin-resistant lactobacilli, *L. rhamnosus* L34 (LR-L34) and *L.casei* L39 (LC-L39), suppressed the secretion and transcription of IL-8 without inhibiting *C. difficile* viability or toxin production. Conditioned media from LR-L34 suppressed the activation of phospho-NF-κB with no effect on phospho-c-Jun. However, LC-L39 conditioned media suppressed the activation of both phospho-NF-κB and phospho-c-Jun. Conditioned media from LR-L34 and LC-L39 also decreased the production of *C. difficile*-induced GM-CSF in HT-29 cells. Immunomodulatory factors present in the conditioned media of both LR-L34 and LC-L39 are heat-stable up to 100°C and > 100 kDa in size.

**Conclusions:**

Our results suggest that *L. rhamnosus* L34 and *L. casei* L39 each produce factors capable of modulating inflammation stimulated by *C. difficile*. These vancomycin-resistant *Lactobacillus* strains are potential probiotics for treating or preventing CDAD.

## Background

*Clostridium difficile* is a gram-positive, spore-forming anaerobe that causes antibiotic-associated diarrhea, colitis, and pseudomembranous colitis in humans [1,2]. *C. difficile*-associated disease (CDAD) is acquired in association with the disruption and alteration of the gut microbiota [3]. The frequency and severity of primary CDAD are increasing; as well as recurrent cases and infections refractory to standard antibiotic therapy [4]. *C. difficile* toxins, toxin A (TcdA, 308 kDa) and toxin B (TcdB, 270 kDa), are the main virulence factors contributing to intestinal tissue damage and severe inflammation [5]. Both toxins disrupt the actin cytoskeleton and tight junctions of intestinal epithelial cells [6,7], and cause apoptotic and necrotic cell death [8,9]. *C. difficile* toxins, TcdA and TcdB, induce the release of chemokines, like IL-8, from intestinal epithelial cells [10-12]. In addition to IL-8, TcdA induces human epithelial cells to secrete other CXC chemokines, including growth-related oncogene (GRO)-α and neutrophil activating protein-78 (ENA-78), along with the CC chemokine, monocyte chemoattractant protein (MCP)-1 [12]. The disruption of tight junctions is thought to enable TcdA and TcdB to enter the laminar propria and submucosa to induce immune cells to secrete chemokines, proinflammatory cytokines, and mediators which promote proinflammatory and cytotoxic effects [4,13]. Proinflammatory cytokines, especially IL-1β, also act on epithelial cells to increase IL-8 secretion and upregulate intercellular adhesion molecule-1 (ICAM-1) expression, leading to increased ICAM-1 and neutrophil CD11/CD18 receptor-dependent neutrophil adhesion [14].

A prominent feature of CDAD results from the release of IL-8 from intestinal epithelial cells that causes a massive influx of neutrophils into the colonic mucosa [4,5,15]. Neutrophil-derived inflammatory mediators exert toxic effects on epithelial cells, causing congestion and edema of the mucosa and epithelial cell damage [14,16]. IL-8 is a major CXC chemokine that mediates neutrophil recruitment, activation, and adhesion. IL-8 potency relies on high binding affinity for neutrophil surface receptors, CXCR1 and CXCR2; which activate chemotaxis [17,18] and robust effector functions [19,20], and trigger the upregulation of adhesion molecules CD11/CD18 that facilitate transendothelial migration and subsequent tissue infiltration [14,21].

CDAD is most often associated with the disruption of a healthy intestinal microbiome after the administration of antibiotics [3]. Potential biotherapies for the treatment and prevention of CDAD are probiotics. In particular, candidate probiotics are capable of enhancing microbiome stability, interfering with pathogen activity, modulating the immune system, and are intrinsically resistant to broad-spectrum antibiotics [22,23]. Meta-analyses of randomized controlled trials [24,25] support the efficacy of probiotic *Saccharomyces boulardii* and *Lactobacillus rhamnosus* GG in the prevention of CDAD. Placebo-controlled clinical trials have shown that lyophilized *S. boulardii* decreased diarrhea associated with β-lactam antibiotics [26], and when used in combination with metronidazole or vancomycin, *S. boulardii* significantly reduced recurrent episodes in patients with CDAD [27,28]. The probiotic effects of *S. boulardii* resulted from a secreted protease that interferes with receptor binding of *C. difficile* toxins A and B to brush borders of human colonic epithelial cells [29] and the downstream modulation of host mitogen-activated protein kinase (MAPK) signaling pathway [30]. *In vitro* studies with probiotic bacteria show that *Lactobacillus delbrueckii* ssp. *bulgaricus* B-30892 inhibits the cytotoxic effects and adhesion of *C. difficile* to Caco-2 cells [31], while specific strains of *Bifidobacterium* spp*.* and *Lactobacillus* spp. have antagonistic effects on the production of *C. difficile* toxins A and B [32].

In the present study, we hypothesized that specific strains of *Lactobacillus* species isolated from healthy hosts produce factors that suppress toxigenic *C. difficile*-induced IL-8 production. We investigated the probiotic effect of 34 *Lactobacillus* infant-fecal isolates on the suppression of IL-8 production from colonic epithelial cells stimulated by *C. difficile*. Conditioned media of two vancomycin-resistant isolates, *L. rhamnosus* L34 and *L. casei* L39, suppressed IL-8 production at the level of transcription without inhibiting *C. difficile* growth or toxin production. Large (>100 kDa) heat-stable, soluble factors are suggested to be responsible for the observed IL-8 suppression.

## Results

### Human-derived *Lactobacillus* spp. produce factors that inhibit IL-8 and GM-CSF production by *C. difficile*-stimulated colonic epithelial cells

**Figure 1:**
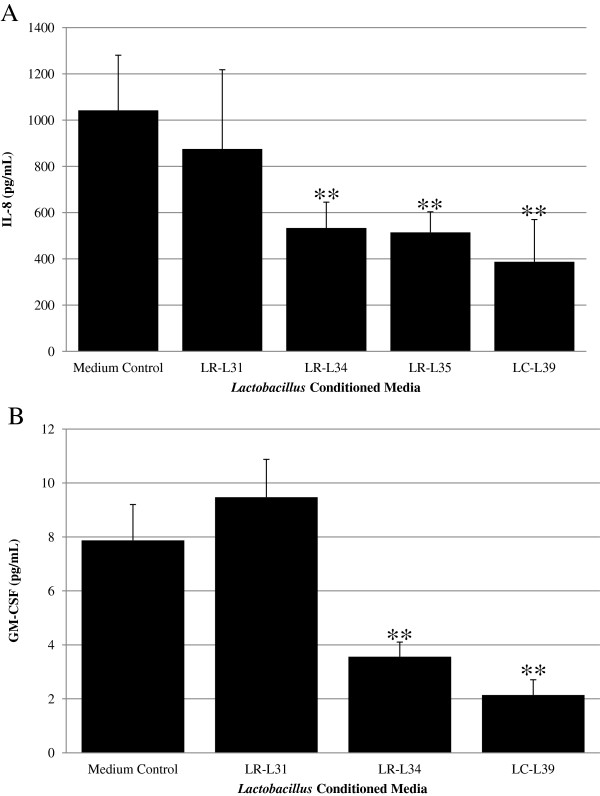
**Infant feces-derived *Lactobacillus *spp. produce factors that suppress pro-inflammatory cytokine production by *C. difficile*-stimulated HT-29 intestinal epithelial cells.** LCM from specific strains of human-derived lactobacilli were found to significantly inhibit IL-8 production by HT-29 cells stimulated with *C. difficile*. Cells were stimulated with *C. difficile* in the presence of LCM for 24 h. **(A)** IL-8 production was monitored by ELISA, and **(B)** GM-CSF production was monitored by a Luminex premixed cytokine assay by Millipore. The results were from three independent experiments in triplicate for figure **(A)** and one experiment in triplicate for figure **(B)** and are expressed as the mean ± SEM, **p-value < 0.01 as compared to medium control.

*Lactobacillus* conditioned media (LCM) from thirty-four human-derived *Lactobacillus* isolates (Additional file 1) were screened for the ability to suppress pro-inflammatory cytokine production from *C. difficile*-stimulated colonic epithelial cells. LCM from 3 of 34 *Lactobacillus* isolates, *L. rhamnosus* L34 (LR-L34), *L. rhamnosus* L35 (LR-L35), and *L. casei* L39 (LC-L39), significantly suppressed IL-8 production by approximately 50% or greater when compared to media control (Figure 1A). These three IL-8-suppressing isolates did not stimulate IL-8 production when co-cultured with HT-29 cells in the absence of *C. difficile* (data not shown). Only a single isolate of *L. casei* was included in this study (Additional file 1), and the third *L. rhamnosus* isolate in this collection, strain L31, did not suppress IL-8 in the same assay (Figure 1A). In a previous study [33], we sequenced and compared the genomes of *L. rhamnosus* L31, L34, and L35. Due to the fact that LR-L34 and LR-L35 were isolated from the same host, had similar colony morphology, were nearly identical at the nucleotide level and similarly genetically distinct from strain LR-L31, we determined that they were most likely independent isolates of the same strain. Therefore, the remaining experiments in this study were conducted excluding LR-L35.

LCM from LR-L34 and LC-L39 were screened for effects on fourteen additional cytokines in a multi-plex luminex assay. In addition to IL-8, *C. difficile* stimulated GM-CSF production, but affected no other cytokines tested (Additional file 2). GM-CSF is an important pro-inflammatory cytokine that allows neutrophils to persist at sites of inflammation [34,35] and enhances the chemotactic response of neutrophils to IL-8 [36]. In addition to IL-8, LCM from both anti-inflammatory lactobacilli strains significantly inhibited the production of GM-CSF (Figure 1B). LCM from a different strain of a matched species of *L. rhamnosus*, strain L31, not only had no effect on IL-8 production, but also did not inhibit GM-CSF production in the same assay (Additional file 2).

Further characterization of isolates LR-L34 and LC-L39 showed that they are resistant to both vancomycin and metronidazole (both with MIC > 256 μg/mL), two drugs commonly used to treat *C. difficile* infection in humans [37,38]. The anti-inflammatory effect of soluble factors produced by LR-L34 and LC-L39 is not unique to HT-29 cells as LCM from both strains also suppresses IL-8 production greater than 50% from human colonocytes (Caco-2) stimulated by *C. difficile* (Additional file 3). Trypan blue dye exclusion indicated that HT-29 and Caco-2 cell viability (>90%) was not compromised by the presence of any LCM tested (data not shown). Furthermore, neither viability of (Additional file 4) nor toxin production by (Additional file 5) *C. difficile* was negatively affected by the presence of LCM. All further *in vitro* experimentation was carried out using the *C. difficile*-HT-29 cell co-culture model.

### *L. rhamnosus* L34 and *L. casei* L39 affect IL-8 gene transcription through decreased activity of NF-κB and c-Jun

**Figure 2:**
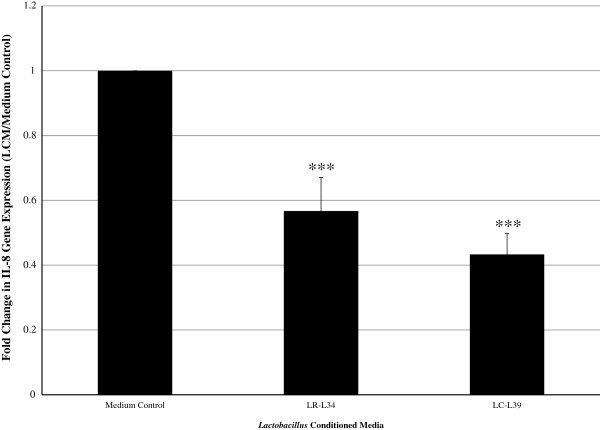
***Lactobacillus *soluble factors suppress IL-8 transcription in HT-29 cells.** IL-8 gene expression was determined in *C. difficile*-stimulated HT-29 cells after 4 h incubation with medium control or LCM from either LR-L34 or LC-L39. Quantitative real-time PCR was conducted with primers specific to IL-8 and GAPDH transcripts. Gene expression data were normalized to housekeeping gene, GAPDH. Fold change ratios of IL-8 (LCM strain/medium control) from one experiment in triplicate were calculated, and results represent the mean ± SEM, ***p-value < 0.001 as compared to medium control.

The effects of soluble factors produced by LR-L34 and LC-L39 on IL-8 transcription were determined by IL-8 gene-specific quantitative RT-PCR. *C. difficile*-stimulated HT-29 cells were treated with LCM for 4 h prior to total RNA isolation. When compared to media control, treatment with LCM from LR-L34 or LC-L39 resulted in an approximate 0.5-fold down-regulation (*p-*value < 0.001) of IL-8 mRNA concentration relative to *gapdh* (Figure 2).

**Figure 3:**
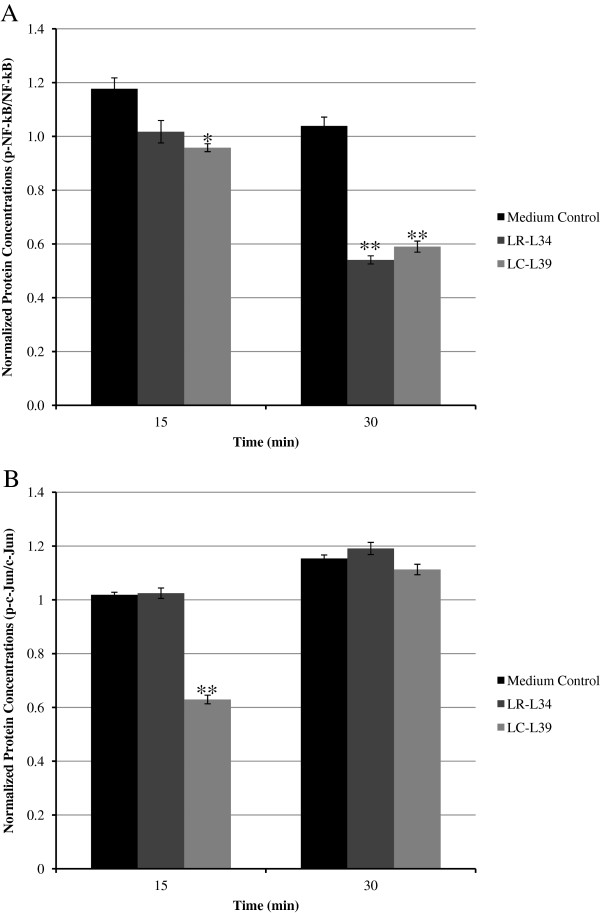
**Human-derived *Lactobacillus *spp. suppress activation of *C. difficile*-induced transcription factors.** Concentrations of activated NF-κB **(A)** and c-Jun **(B)** were determined by western blot on whole cell lysates of HT-29 cells stimulated with *C. difficile* with or without medium control or LCM treatment. Concentrations were measured at 15 and 30 min using antibodies corresponding to p-NF-κB p65, NF-κB p65, β-actin, p-c-Jun, and c-Jun. Relative protein concentrations were determined by densitometry, and activated transcription factors were normalized to their non-activated counterpart (p-NF-κB p65 (Ser 536) to NF-κB p65; p-c-Jun to c-Jun). The results were from three independent experiments in duplicate and are expressed as the mean ± SEM, *p-value <0.05 and **p-value <0.01.

Transcriptional regulation of IL-8 is mediated via NF-κB and AP-1 by the upstream phosphorylation of subunits p65 and c-Jun, which result in the respective activation of NF-κB and AP-1 and subsequent downstream transcription of IL-8 [39-42]. To determine whether or not LR-L34 or LC-L39 produce factors that affect activation of NF-κB or c-Jun, *C. difficile-*stimulated HT-29 cells were treated with LCM from either LR-L34 or LC-L39 for 15 and 30 min, then were assayed for effects on phosphorylated NF-κB (p-NF-κB) and c-Jun (p-c-Jun) concentrations by western blot. LCM from LR-L34 decreased p-NF-κB by 47.93% at 30 min only (*p-*value <0.001, Figure 3A), and did not affect p-c-Jun at either time point (Figure 3B). A 15 min treatment with LC-L39 LCM resulted in decreased concentrations of p-NF-κB (18.62%, *p*-value <0.01, Figure 3A) and p-c-Jun (38.19%, *p*-value <0.001, Figure 3B) while a 30 min treatment resulted in a 43.19% decrease in p-NF-κB (*p*-value <0.001, Figure 3A).

### The anti-inflammatory factor(s) produced by *L. rhamnosus* L34 and *L. casei* L39 is heat-stable and greater than 100 kDa in size

**Figure 4:**
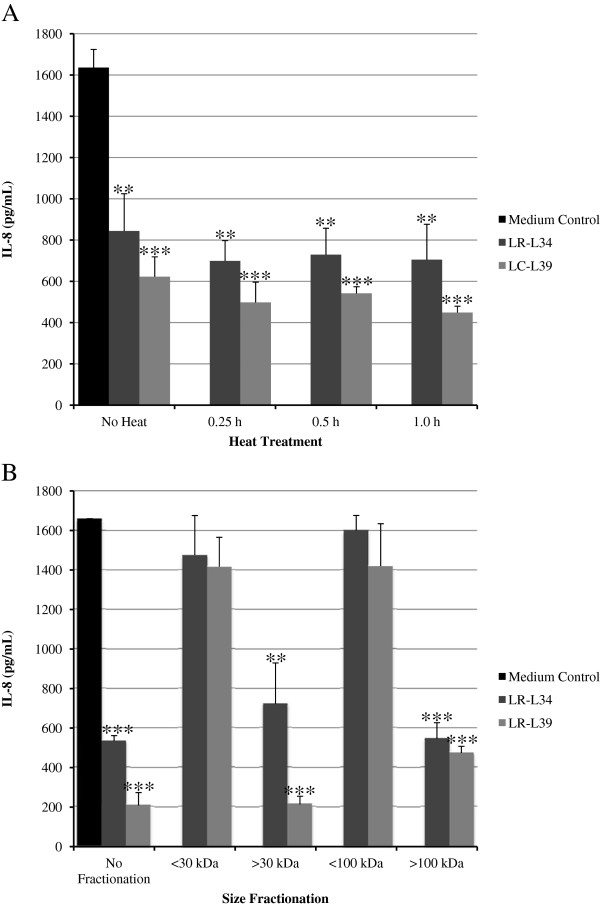
**IL-8 suppression by LCM after heat treatment and size fractionation.** Soluble factors in LCM from LR-L34 and LC-L39 were assayed for heat stability and size prediction. LCM from *L. rhamnosus* L34 and *L. casei* L39 was either heated to boiling for various time points **(A)** or size fractionated by filtration **(B)** and the effects on IL-8 production by *C. difficile*-stimulated HT-29 cells was evaluated by ELISA. The results were from three independent experiments in triplicate and are expressed as the mean ± SEM, **p-value <0.01 and ***p-value <0.001.

Heat tolerance and size prediction of anti-inflammatory factors in LCM from LR-L34 and LC-L39 were characterized as follows. LCM from LR-L34 and LC-L39 were heated to 100°C for 15 min, 30 min, and 1 h and then tested for the ability to suppress *C. difficile*-induced IL-8 production in HT-29 cells. The ability to suppress IL-8 production in this assay was retained at all time points (Figure 4A). Size fractionation of LR-L34 and LC-L39 LCM was performed using 30 kDa and 100 kDa Amicon® Centrifugal Filters and filtrates containing factors <30 kDa and <100 kDa did not suppress IL-8 activity, while fractions containing factors >100 kDa retained the anti-IL-8 activity in each case (Figure 4B).

## Discussion

Standard therapy for treating CDAD with metronidazole and vancomycin is effective, but the association of these drugs with high relapse rates represents a major health problem [43-45]. Recent reports estimate the rate of recurrence after an initial episode of CDAD to be 13-38% [46-49]. Standard therapy for recurrent CDAD has been modified to include probiotics like *L.rhamnosus* GG, *L. plantarum* 299v, or *S. boulardii* which reduce the rate of recurrence around 20-30% [28,50,51]. Health promoting effects of lactobacilli include the stabilization of indigenous microbial populations, protection against intestinal infection, modulation of the immune system, and effects on gene expression in the human mucosa [52-54]. In addition, a recent systematic review and meta-analysis indicated that probiotics given concurrently with antibiotics reduced the risk of antibiotic-associated diarrhea and *C. difficile* infection [55]. A key characteristic of the pathophysiology of CDAD is an inflammatory response with a marked neutrophil accumulation resulting from secreted IL-8 [14-16,21]. Lactobacilli are known to inhibit IL-8 production by intestinal epithelial cells stimulated with bacterium-derived LPS [56] but have not yet been associated with the modulation of *C. difficile*-induced IL-8 production.

We identified two *Lactobacillus* isolates from a library of infant feces-derived *Lactobacillus* spp. that can significantly suppress IL-8 production by *C. difficile*-stimulated HT-29 cells. IL-8 suppression by LR-L34 and LC-L39 did not result from negative effects on *C. difficile* viability as colony counts from the co-culture supernatants of either *C. difficile* alone, or in combination with MRS, or LCM from LR-L34 or LC-L39, were not significantly different (Additional file 4). Trejo *et al*. [32] demonstrated that spent culture supernatants of *B. bifidum* 5310 and *L. plantarum* 83114 diminished the production of toxin A and toxin B by *C. difficile* ATCC 9689 and clinical isolate 117. IL-8 suppression by LR-L34 and LC-L39 seems not to result from attenuated toxin production and endocytosis by HT-29 cells. No significant difference of intracellular toxin concentrations in *C. difficile* and HT-29 cells was seen by LCM treatment as compared to medium control (Additional file 5). However, we cannot exclude the possibility that LCM of LR-L34 and LC-L39 interfere with toxin self-cleavage in the endosome prior to entering the cytosol which induces several downstream inflammatory consequences [57]. It is likely that *C. difficile*-secreted toxins were endocytosed by HT-29 cells as a very small amount of toxin was present in tissue culture medium at 24 h of the co-culture assay.

LR-L34 and LC-L39 suppressed IL-8 gene transcription at 4 h after co-culture with HT-29 cells which is in agreement with a report of Imaoka *et al*. [58] for *B. bifidum* strain Yakult. The IL-8 gene promoter contains binding sites for transcription factors such as NF-κB and AP-1 [59], which control the transcription of IL-8 in *C. difficile*-stimulated HT-29 colonic epithelial cells [40,60]. LCM from LR-L34 decreased p-NF-κB, and did not affect p-c-Jun while LC-L39 LCM decreased both of p-NF-κB and p-c-Jun, with greater effects on pNF-κB. Modulation of pro-inflammatory signaling pathways by probiotic bacteria and yeasts have been demonstrated previously. Ma *et al*. [61] reported that *L. reuteri* inhibited TNF-induced IL-8 production in both T84 and HT-29 intestinal epithelial cells by inhibiting nuclear translocation of NF-κB. *S. boulardii* modifies host cell pro-inflammatory signaling pathways during bacterial infection by blocking the activation of NF-κB and MAPK [62-64]. Our data showed that *C. difficile* stimulated GM-CSF production in addition to IL-8 and conditioned media generated from LR-L34 and LC-L39 can also suppress GM-CSF production. The ability to suppress GM-CSF of these lactobacilli potentially enhances their anti-inflammatory effects on *C. difficile* infection.

Probiotic factors can vary from organism to organism in regards to activity, size, and stability. Sougioultzis *et al.*[65] reported that *S. boulardii* probiotic yeast produce a heat stable, <1 kDa soluble, anti-inflammatory compound that blocks NF-κB activation and NF-κB-mediated IL-8 gene expression in both HT-29 colonic epithelial cells and THP-1 monocytes. *L. rhamnosus* GG secretes p40 (40 kDa) and p75 (75 kDa) proteins that activate EGFR and downstream PI3K/Akt and PKC signaling that modulate intestinal epithelial cell survival and growth [66]. Castagliuolo *et al.*[29] reported that a 54-kDa protease of *S. boulardii* can inhibit the effect of *C. difficile* toxins A and B in HT-29 cells. *L. reuteri* ATCC PTA 6475 produces the small molecule, histamine, which inhibits TNF production in THP-1 monocytes via PKA and ERK signaling [67]. Active substances in LCM of LR-L34 and LC-L39 were found to be heat-stable and >100 kDa. Although LR-L34 and LC-L39 also inhibit TNF production by THP-1 monocytes, they do not produce histamine (data not shown). Therefore the TNF-suppressive mechanism of action of these lactobacilli must be different from *L. reuteri*, and further investigation is needed to characterize the soluble factors produced by these specific *Lactobacillus* isolates. The discovery of vancomycin-resistant LR-L34 and LC-L39 with IL-8-suppressing ability in this study offers us potential probiotic strains for combating CDAD. These strains may harbor other probiotic properties, including the ability to replenish normal microbiota, a key factor for treating CDAD as shown by the recent successes with intestinal microbiome transplant therapies [68,69].

## Conclusions

We have demonstrated the probiotic effect of two vancomycin-resistant strains, *L. rhamnosus* L34 and *L. casei* L39, on the suppression of IL-8 production from *C. difficile*-stimulated colonic epithelial cells. These strains suppressed IL-8 production by inhibiting activation of transcription factors for IL-8 gene expression without inhibiting *C. difficile* growth or toxin production. Our data also suggest that heat-stable, >100 kDa factors are responsible for IL-8 suppression. These vancomycin-resistant *Lactobacillus* strains are potential probiotics for treating or preventing CDAD.

## Methods

### Bacterial strains and culture conditions

Thirty-four *Lactobacillus* spp. isolated from infant feces were analyzed in this study (Additional file 1). All lactobacilli were routinely cultured in an anaerobic chamber (Concept Plus, Ruskinn Technology, UK) (10% CO_2_, 10% H_2_, and 80% N_2_) for 24 h at 37°C in de Man, Rogosa, Sharpe (MRS) medium (Oxoid, England).

A *C. difficile* isolate, designated strain B2-CU-0001-54 was obtained from feces of an infected patient positive for *C. difficile* toxins A and B by VIDAS® *Clostridium difficile* A & B (Biomérieux, France) at the Department of Microbiology, Faculty of Medicine, Chulalongkorn University. This strain is positive for TcdA and TcdB as determined by PCR for toxin A and B genes [70] and the reactivity with mouse anti-TcdA and anti-TcdB monoclonal antibodies (Meridian Life Science, Inc.). *C. difficile* B2-CU-0001-54 was routinely cultured anaerobically on Brucella agar (Oxoid, England) at 37°C for 48 h. Cells were harvested, re-suspended in McCoy’s medium, and adjusted to a McFarland 6 standard (1.8×10^9^ cells/mL) prior to co-culture with HT-29 cells. This study was approved by the Ethics Committee of Faculty of Medicine, Chulalongkorn University, Bangkok, Thailand (COA no.617/2011, IRB no.246/54). Written informed parental consent for fecal samples was obtained from participants.

### Minimum inhibitory concentration assay

The minimum inhibitory concentration of vancomycin on LR-L34 and LC-L39 was determined by the broth microdilution procedure as previously described [71]. Briefly, 100 μl of lactobacilli (final concentration 1 × 10^6^ CFU/mL or 1 × 10^5^ CFU/well) were inoculated into the wells of a 96 well plate containing 100 μl of vancomycin in serial 2-fold dilutions from 256 to 0.125 μg/mL, then were cultured anaerobically for 48 h. Optical density was measured at 600 nm using a microplate reader Multiskan® EX (Thermo Scientific, USA). The results were compared with growth control (lactobacilli alone) and the endpoint of MIC is the concentration where no growth or a reduction in growth by 90%, is observed.

### Preparation of *Lactobacillus* conditioned media

LCM were prepared as previously described [72]. Briefly, 24 h cultures were adjusted to an OD_600_ 0.1 and incubated anaerobically for 48 h. Supernatants were collected, filtered with 0.22 μm Millex-GV Filter Units (Millipore, USA), and 500 μL aliquots were concentrated by Eppendorf Vacufuge® vacuum concentrator (Eppendorf North America, USA) at 60°C for 2.5 h. Pellets were resuspended in an equal volume of McCoy’s 5a modified medium (Gibco-Invitrogen, Carlsbad, CA, USA) or Eagle’s Minimal Essential Medium (Gibco-Invitrogen, USA) and stored at −20°C until further analysis.

### Cell lines and culture conditions

Human colonic epithelial cells, HT-29 or Caco-2, were obtained from the American Type Culture Collection (ATCC HTB-38 and HTB-37, respectively; Manassas, VA, USA). HT-29 cells were maintained in McCoy’s 5a modified medium supplemented with 10% (v/v) heat-inactivated fetal bovine serum (Gibco-Invitrogen, USA) at 37°C under 5% CO_2_ for 48 h. Caco-2 cells were maintained in Eagle’s Minimal Essential Medium supplemented with 20% (v/v) heat-inactivated fetal bovine serum at 37°C under 5% CO_2_ for 72 h. Adherent cells were detached with 0.25% (v/v) Trypsin (Gibco-Invitrogen, USA) in 1 mM EDTA (Gibco-Invitrogen, USA) and resuspended in their respective medium. Resuspensions of each cell type were used in subsequent *C. difficile* co-culture assays.

### LCM treatment and *C. difficile* co-culture with colonic epithelial cells

Colonic epithelial cells were treated with LCM and stimulated to produce IL-8 by co-culture with *C. difficile*. HT-29 (2.0 × 10^4^ cells) or Caco-2 cells (5.0 × 10^4^ cells) were pre-incubated for 24 h in a 96-well format as described above. LCM (5% v/v) was added with or without the subsequent addition of viable *C. difficile* B2-CU-0001-54 (6.0 × 10^6^ CFU/well with HT-29 cells or 4.5 × 10^7^ CFU/well with Caco-2 cells) and co-incubated for an additional 24 h. Cell culture supernatants were collected by centrifugation (125 × g, 4°C for 7 min) and stored at -20°C until further use.

### Effects of LCM on *C. difficile* viability

To ensure IL-8 suppression did not result from the antagonism of *C. difficile* growth by soluble factors in LCM, *C. difficile* B2-CU-0001-54 was assayed for viability after co-incubation with LCM and HT-29 cells. Briefly, co-culture supernatants were serially diluted and cultured anaerobically on Brucella agar (Oxoid, England) at 37°C for 48 h. Counts of isolated *C. difficile* B2-CU-0001-54 colonies from co-culture assays with and without LCM treatment were compared.

### Effects of LCM on *C. difficile* toxins in the co-culture assay

To determine whether *Lactobacillus* spp. produce factors that suppress *C. difficile* toxin secretion, intracellular and secreted toxin concentrations from co-culture assays were determined as previously described [32] with the following modifications. Secreted toxin was measured from LCM-treated, *C.difficile*-stimulated HT-29 cell supernatants, which were concentrated 10-fold by speed vacuum drying and spotted onto polyvinylidene fluoride (PVDF) membranes (Bio-Rad, Philadelphia, USA). Blocked membranes were incubated in succession with mouse anti-TcdA or anti-TcdB monoclonal antibodies (Meridian Life Science, Inc.), biotinylated goat anti-mouse IgG, and extravidin-alkaline phosphatase (Sigma-Aldrich, St. Louis, MO, USA). The presence of toxin was determined colorimetrically with nitro blue tetrazolium/5-bromo-4-chloro-3-indolyl-phosphate (NBT/BCIP) solution (Sigma-Aldrich, St. Louis, MO, USA). Intracellular toxin concentrations were determined from LCM-treated, *C.difficile*-stimulated HT-29 cell pellets. Pellets from 20 ml of cell culture were washed, resuspended in 2 ml cell culture medium, and lysed by sonication (Vibra-Cell™, Sonics & Materials, Inc., USA). Cell lysates were collected, 100 μl spotted onto PVDF membranes, and analyzed for the presence of toxin as described above. Toxin concentrations were calculated by ChemiDoc™ XRS (Bio-Rad, Philadelphia, USA).

### Effects of LCM on cytokine production by ELISA and Luminex assays

Supernatants from co-culture assays were tested for the effects of soluble factors produced by *Lactobacillus* on IL-8 production and other related cytokines. IL-8 concentrations in LCM-treated, *C. difficile*-stimulated HT29 or Caco-2 cell co-culture supernatants were measured using a Human CXCL8/IL-8 ELISA kit (R&D Systems, Minneapolis, MN) according to the manufacturer’s instructions. To determine whether *Lactobacillus* spp. can modulate cytokines other than IL-8 in this assay, cell supernatants from *C. difficile*-stimulated HT-29 cells in the presence or absence of LCM were screened by a Human Cytokine/Chemokine-Premixed 14-plex kit (Millipore, Billerica, MA) in a Luminex 100 system (Luminex Corporation, Austin, TX) for quantification of the analytes GM-CSF, IFN-γ, IL-1β, IL-2, IL-4, IL-5, IL-6, IL-7, IL-8, IL-10, IL-12 (p70), IL-13, MCP-1, and TNF-α. Analytes at concentrations exceeding the minimum detectable dose were evaluated and raw data were obtained with MasterPlex CT version 1.2.0.7 and analyzed with MasterPlex QT version 5.0.0.73 (Hitachi MiraiBio, San Francisco, CA).

### Analysis of IL-8 gene transcription by qPCR

The effects of LCM on the transcription of IL-8 were determined by qPCR as previously described [58] with the following modifications. HT-29 cells (8.0×10^5^ cells) were pre-incubated in a 24-well format as outlined above. Cells were LCM-treated (5% v/v) with or without the subsequent addition of viable *C. difficile* B2-CU-0001-54 (2.4×10^8^ CFU/well) at 37°C, 5% CO_2_ for 4 h. Total RNA was extracted from HT-29 cells using TRIzol reagent (Invitrogen, USA) according to the manufacturer’s instructions. Synthesis of cDNA was completed using the SuperScript® VILO™ cDNA Synthesis kit (Invitrogen), and qPCR was performed in a LightCycler® 2.0 (Roche, Germany) for 45 cycles of: 15 s at 94°C, 25 s at 60°C, and 25 s at 72°C. The amplified product was detected using Light Cycler® Fast Start DNA Master^PLUS^ SYBR Green I (Roche, Germany) at 530 nm. The following primers were used to amplify cDNA fragments: IL-8 forward primer (5′-ACACTGCGCCAACACAGAAATTA-3′), IL-8 reverse primer (5′-TTTGCTTGAAGTTTCACTGGCATC-3′); GAPDH forward primer (5′-GCACCGTCAAGGCTGAGAAC-3′), GAPDH reverse primer (5′-ATGGTGGTGAAGACGCCAGT-3′). IL-8 gene expression, relative to GAPDH, was calculated according to the 2^-ΔΔCp^ method [73].

### Examination of cell signaling pathways by quantitative western blot

Changes in the NF-κB signaling pathway coordinating with LCM treatment were analyzed by western blot as previously described [30] with minor modifications. LCM-treated, *C. difficile-*stimulated HT-29 cells were lysed in a radioimmunoprecipitation assay (RIPA) buffer (50 mM TrisHCl, pH7.4, 150 mM NaCl, 10% NP-40, 0.5% Na-DOC, 0.1% SDS). Protein concentrations from cell lysates were measured using the Pierce® BCA protein assay kit (Pierce Biotechnology, Illinois, USA). Cell extracts were fractionated by 10% sodium dodecyl sulfate polyacrylamide gel electrophoresis (SDS-PAGE), transferred onto PVDF membranes (Bio-Rad, Philadelphia, USA), and blocked (10% non-fat milk in TBST (50 mM Tris, pH 7.5, 0.15 M NaCl, 0.05% Tween 20). Blocked membranes were incubated with mouse antibodies against NF-κB (p65), phospho-NF-κB (p65), c-Jun, phospho-c-Jun and β-actin (Santa Cruz Biotechnology, California, USA), washed with TBST and treated with horseradish peroxidase-labeled goat anti-mouse secondary antibodies for 1 h. Peroxidase signals were detected and imaged by ChemiDoc™ XRS (Bio-Rad, Philadelphia, USA).

### Characterization of IL-8 suppressive factors in LCM

*Lactobacillus* anti-inflammatory factors responsible for suppressing IL-8 production from *C. difficile*-stimulated colonic epithelial cells were assessed for thermal stability and size estimation. Heat stability was assessed by heating LCM to 100°C for 15 min, 30 min, and 1 h. The sizes of active factors present in LCM were estimated by 30 kDa and 100 kDaAmicon® Ultra-4 Centrifugal Filters (Merck, Massachusetts, USA). After each treatment, LCM was tested for the ability to suppress IL-8 production from *C. difficile*-treated HT-29 cells using techniques outlined above.

### Statistical analyses

All experiments were performed at least in triplicate and the results were reported as mean ± standard deviation or standard error of mean (SEM). The data were analyzed in Microsoft Excel using the Student’s *t*-test with one-tailed distribution and considered statistically significant at a *p*-value ≤ 0.05, unless otherwise stated.

## Competing interests

The authors declare that they have no competing interests.

## Authors’ contributions

JKS, JV, and ST conceived and designed the study. PB designed and performed the experiments. SV carried out Luminex assay. JKS, JV, and ST supervised and provide funding for the research. PB, JKS, and ST wrote the manuscript. All authors read and approved the final manuscript.

## Supplementary Material

Additional file 1 **Infant feces-derived *Lactobacillus *** **spp. used in this study.** Click here for file

Additional file 2 **Production of cytokines and chemokines by *C. difficile*-stimulated HT-29 cells.** Click here for file

Additional file 3 **Infant feces-derived *Lactobacillus *** **spp. produce factors that suppress pro-inflammatory cytokine production by *C. difficile*-stimulated Caco-2 intestinal epithelial cells.** LCM from human-derived lactobacilli were found to significantly inhibit IL-8 production from Caco-2 cells stimulated with *C. difficile*. Cells were stimulated with *C. difficile* in the presence of LCM for 24 h and IL-8 production was monitored by ELISA. The results were from three independent experiments in triplicate and are expressed as the mean ± SEM, **p-value <0.01 as compared to medium control. Click here for file

Additional file 4 ***C. difficile *** **viability was not affected by *Lactobacillus *** **conditioned media.** IL-8 suppression did not result from LCM effects on the antagonism of *C. difficile* growth. *C. difficile* B2-CU-0001-54 was assayed for viability after co-incubation with LCM and HT-29 cells. Counts of isolated *C. difficile* B2-CU-0001-54 colonies from co-culture assays with and without LCM treatment from three independent experiments in triplicate were compared and results are reported as mean ± SD. No significant difference in viability of *C. difficile* was seen by LCM treatment as compared to medium control. Click here for file

Additional file 5 ***Lactobacillus *** **conditioned media had no effect on *C. difficile *** **toxins in co-culture assay.** IL-8 suppression did not result from attenuated toxin production and endocytosis to HT-29 cells. Extracellular and intracellular toxin concentrations were determined from LCM-treated, *C. difficile*-stimulated HT-29 cells. Cell culture supernatants or lysates of *C. difficile* and HT-29 cells were collected, spotted onto PVDF membranes and assayed in succession with mouse anti-TcdA or anti-TcdB monoclonal antibodies. The presence of toxin was determined colorimetrically and toxin concentrations were calculated by ChemiDoc™ XRS (Bio-Rad, Philadelphia, USA). The results were from three independent experiments in triplicate and are expressed as the mean ± SEM. No significant difference in intracellular toxin concentrations was seen by LCM treatment as compared to medium control. **(A)** Whole cell lysates, **(B)** culture supernatant of HT-29 cells, **(C)** the ratio between spot intensity of *C. difficile* co-culture with LCM and *C. difficile* with MRS. Click here for file
