# Supplementary material for: Lactobacillus rhamnosus L34 and Lactobacillus casei L39 suppress Clostridium difficile-induced IL-8 production by colonic epithelial cells
Source: BMC Microbiol. 2014 Jul 2;14:177. doi: 10.1186/1471-2180-14-177 (PMC4094603; doi:10.1186/1471-2180-14-177)
Supplement: Additional file 1 — Infant feces-derived Lactobacillus spp. used in this study. [file 1471-2180-14-177-S1.docx]

**Additional file 1: Infant feces-derived *Lactobacillus* spp. used in this study**

| Bacterial Strain | Infant Diet | Vancomycin Susceptibility | NCBI 16 rRNA Gene Sequence Identity (%) |
| --- | --- | --- | --- |
| *L. casei* L39 (LC-L39) | Formula and breast milk | R | 99 |
| *L. fermentum* L7 (LF-L7) | Formula and breast milk | R | 98 |
| *L. fermentum* L9 (LF-L9) | Formula and breast milk | R | 99 |
| *L. fermentum* L12 (LF-L12) | Formula | R | 98 |
| *L. fermentum* L15 (LF-L15) | Formula and breast milk | R | 98^a^ |
| *L. fermentum* L18 (LF-L18) | Breast milk | R | 95 |
| *L. fermentum* L21 (LF-L21) | Formula and breast milk | R | 98 |
| *L. fermentum* L24 (LF-L24) | Formula and breast milk | R | 98^a^ |
| *L. gasseri* L1 (LG-L1) | Formula | S | 99 |
| *L. gasseri* L2 (LG-L2) | Formula and breast milk | S | 99 |
| *L. gasseri* L5 (LG-L5) | Formula, breast milk, banana | S | 99 |
| *L. gasseri* L10 (LG-L10) | Formula and breast milk | S | 93 |
| *L. gasseri* L20 (LG-L20) | Formula | S | 95 |
| *L. gasseri* L25 (LG-L25) | Formula and breast milk | S | 96 |
| *L. gasseri* L26 (LG-L26) | Formula and breast milk | S | 93 |
| *L. gasseri* L29 (LG-L29) | Formula and breast milk | S | 89 |
| *L. gasseri* L30 (LG-L30) | Breast milk | S | 99 |
| *L. gasseri* L38 (LG-L38) | Breast milk and banana | S | 89 |
| *L. gasseri* L41 (LG-L41) | Formula and breast milk | S | 96 |
| *L. mucosae* L14 (LM-L14) | Formula and breast milk | R | 97 |
| *L. oris* L27 (LO-L27) | Formula, breast milk, banana | R | 98 |
| *L. rhamnosus* L31 (LR-L31) | Breast milk | R | 100 |
| *L. rhamnosus* L34 (LR-L34) | Breast milk | R | 99 |
| *L. rhamnosus* L35 (LR-L35) | Breast milk | R | 99 |
| *L. ruminis* L13 (LRU-L13) | Breast milk | R | 99 |
| *L. ruminis* L28 (LRU-L28) | Formula, breast milk, banana | R | 92 |
| *L. salivarius* L6 (LS-L6) | Formula and breast milk | R | 98 |
| *L. salivarius* L8 (LS-L8) | Formula and breast milk | R | 98 |
| *L. salivarius* L11 (LS-L11) | Formula and breast milk | R | 99 |
| *L. salivarius* L17 (LS-L17) | Breast milk | R | 94 |
| *L. salivarius* L22 (LS-L22) | Formula and breast milk | R | 94 |
| *L. salivarius* L23 (LS-L23) | Formula and breast milk | R | 99 |
| *L. salivarius* L40 (LS-L40) | Formula and breast milk | R | 99 |
| *L. vaginalis* L19 (LV-L19) | Formula | R | 98 |

^a^ Sequence for these isolates also matched *L. mucosae* 16S rDNA at 98%.
