# Supplementary material for: Lactobacillus rhamnosus L34 and Lactobacillus casei L39 suppress Clostridium difficile-induced IL-8 production by colonic epithelial cells
Source: BMC Microbiol. 2014 Jul 2;14:177. doi: 10.1186/1471-2180-14-177 (PMC4094603; doi:10.1186/1471-2180-14-177)
Supplement: Additional file 2 — Production of cytokines and chemokines by C. difficile -stimulated HT-29 cells. [file 1471-2180-14-177-S2.docx]

**Additional file 2. Production of cytokines and chemokines by *C. difficile*-stimulated HT-29 cells**

| Cytokine/Chemokine | No *C. difficile* | | *C. difficile* | | | | | | | |
| --- | --- | --- | --- | --- | --- | --- | --- | --- | --- | --- |
|  |  |  | Media control | | LR-L31 | | LR-L34 | | LR-L39 | |
|  | pg/ml | SD | pg/ml | SD | pg/ml | SD | pg/ml | SD | pg/ml | SD |
| IL-8 | 64.835 | 2.454 | 347.430 | 42.016 | 414.253 | 47.472 | 59.067 | 10.380 | 37.587 | 9.552 |
| GM-CSF | 3.865 | 0.742 | 7.875 | 1.322 | 9.473 | 1.400 | 3.560 | 0.541 | 2.143 | 0.561 |
| IFN-γ | 0.290 | 0.000 | 0.060 | 0.000 | 0.110 | 0.087 | 0.000 | 0.000 | 0.000 | 0.000 |
| IL-4 | 6.470 | 8.881 | 6.160 | 0.000 | 5.567 | 2.260 | 0.450 | 0.779 | 2.080 | 3.080 |
| IL-6 | 1.835 | 0.021 | 1.820 | 0.000 | 1.827 | 0.015 | 1.847 | 0.031 | 1.817 | 0.015 |
| IL-7 | 0.000 | 0.000 | 1.800 | 0.000 | 2.250 | 1.587 | 0.000 | 0.000 | 0.000 | 0.000 |
| IL-10 | 1.535 | 0.078 | 1.480 | 0.000 | 1.513 | 0.029 | 1.450 | 0.030 | 1.403 | 0.029 |
| IL-12(p70) | 5.105 | 0.049 | 5.070 | 0.000 | 5.160 | 0.191 | 5.010 | 0.017 | 4.973 | 0.023 |
| IL-13 | 4.170 | 0.255 | 3.990 | 0.255 | 4.170 | 0.238 | 4.007 | 0.029 | 3.810 | 0.000 |
| MCP-1 | 3.980 | 0.042 | 3.920 | 0.042 | 3.890 | 0.030 | 3.910 | 0.035 | 3.880 | 0.017 |
| TNF-α | 0.580 | 0.014 | 0.610 | 0.000 | 0.657 | 0.006 | 0.730 | 0.017 | 0.603 | 0.047 |

*These results were obtained from a single experiment in triplicate and are expressed as the mean ± SD.
