# Supplementary figures and images for: Lactobacillus rhamnosus L34 and Lactobacillus casei L39 suppress Clostridium difficile-induced IL-8 production by colonic epithelial cells
Source: BMC Microbiol. 2014 Jul 2;14:177. doi: 10.1186/1471-2180-14-177 (PMC4094603; doi:10.1186/1471-2180-14-177)

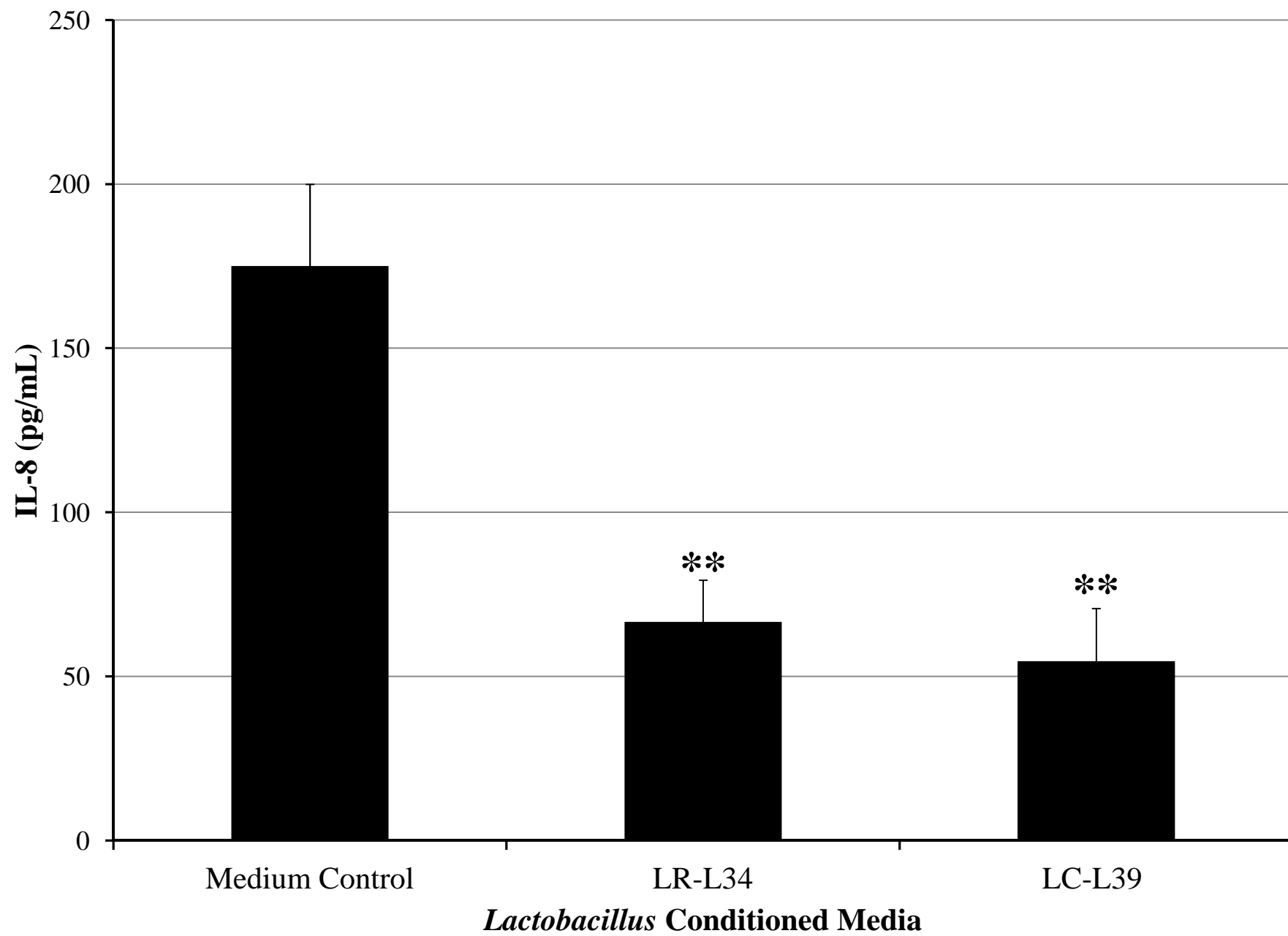

Supplement: Additional file 3 — Infant feces-derived Lactobacillus spp. produce factors that suppress pro-inflammatory cytokine production by C. difficile -stimulated Caco-2 intestinal epithelial cells. LCM from human-derived lactobacilli were found to significantly inhibit IL-8 production from Caco-2 cells stimulated with C. difficile. Cells were stimulated with C. difficile in the presence of LCM for 24 h and IL-8 production was monitored by ELISA. The results were from three independent experiments in triplicate and are expressed as the mean ± SEM, **p-value <0.01 as compared to medium control. [file 1471-2180-14-177-S3.pdf]

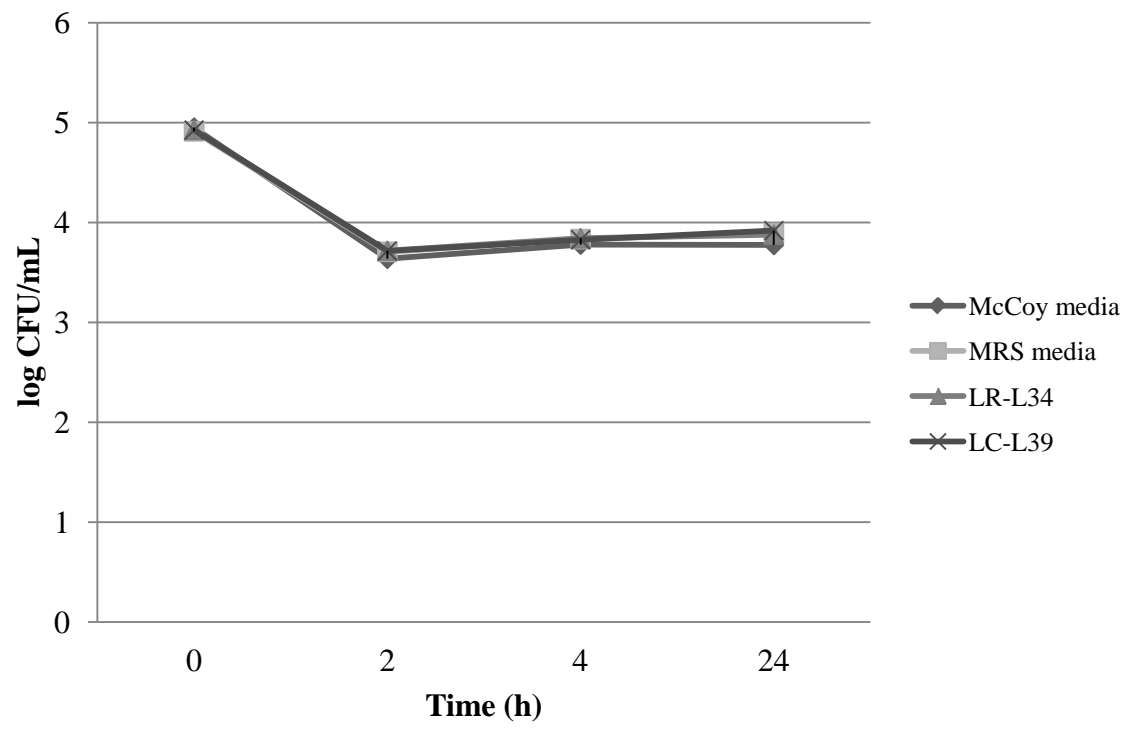

Supplement: Additional file 4 — C. difficile viability was not affected by Lactobacillus conditioned media. IL-8 suppression did not result from LCM effects on the antagonism of C. difficile growth. C. difficile B2-CU-0001-54 was assayed for viability after co-incubation with LCM and HT-29 cells. Counts of isolated C. difficile B2-CU-0001-54 colonies from co-culture assays with and without LCM treatment from three independent experiments in triplicate were compared and results are reported as mean ± SD. No significant difference in viability of C. difficile was seen by LCM treatment as compared to medium control. [file 1471-2180-14-177-S4.pdf]

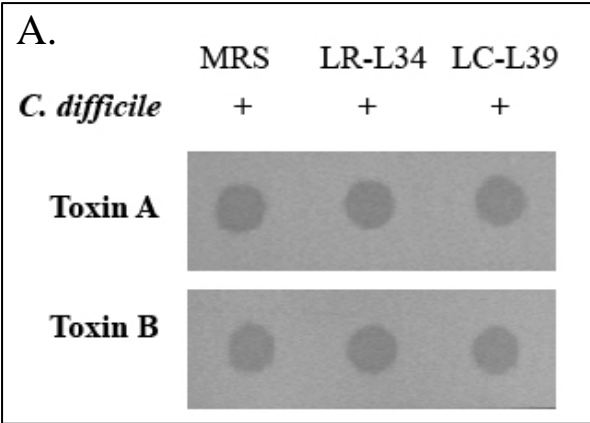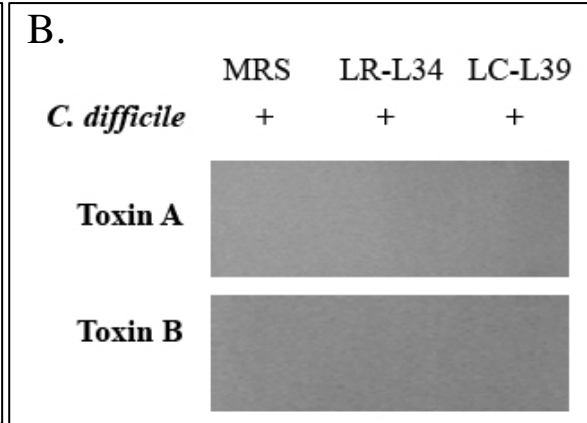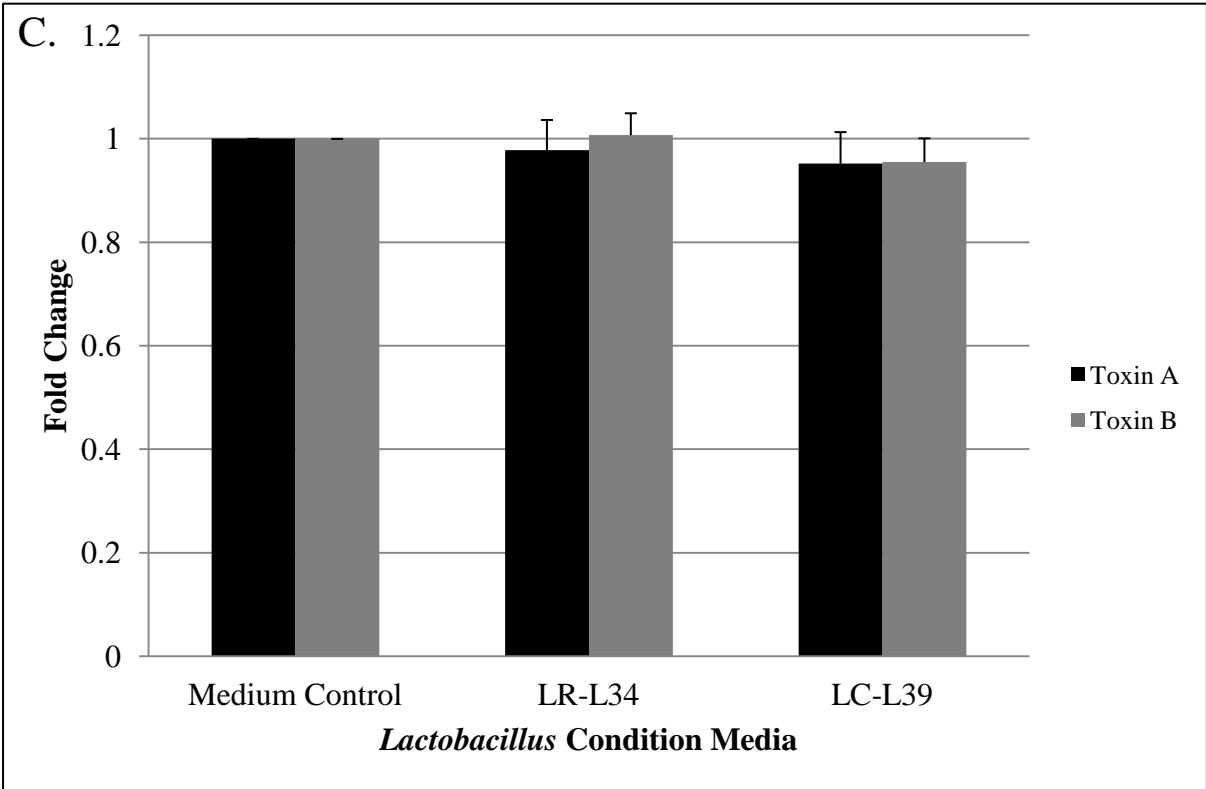

Supplement: Additional file 5 — Lactobacillus conditioned media had no effect on C. difficile toxins in co-culture assay. IL-8 suppression did not result from attenuated toxin production and endocytosis to HT-29 cells. Extracellular and intracellular toxin concentrations were determined from LCM-treated, C. difficile-stimulated HT-29 cells. Cell culture supernatants or lysates of C. difficile and HT-29 cells were collected, spotted onto PVDF membranes and assayed in succession with mouse anti-TcdA or anti-TcdB monoclonal antibodies. The presence of toxin was determined colorimetrically and toxin concentrations were calculated by ChemiDoc™ XRS (Bio-Rad, Philadelphia, USA). The results were from three independent experiments in triplicate and are expressed as the mean ± SEM. No significant difference in intracellular toxin concentrations was seen by LCM treatment as compared to medium control. (A) Whole cell lysates, (B) culture supernatant of HT-29 cells, (C) the ratio between spot intensity of C. difficile co-culture with LCM and C. difficile with MRS. [file 1471-2180-14-177-S5.pdf]
